# Nano–Liposomes Double Loaded with Curcumin and Tetrandrine: Preparation, Characterization, Hepatotoxicity and Anti–Tumor Effects

**DOI:** 10.3390/ijms23126858

**Published:** 2022-06-20

**Authors:** Jia-Wen Song, Yu-Shi Liu, Yu-Rou Guo, Wen-Xiao Zhong, Yi-Ping Guo, Li Guo

**Affiliations:** 1 State Key Laboratory of Southwestern Chinese Medicine Resources, Chengdu University of Traditional Chinese Medicine, Chengdu 611137, China; songjiawen@stu.cdutcm.edu.cn (J.-W.S.); liuyushi@stu.cdutcm.edu.cn (Y.-S.L.); 2 School of Pharmacy, Chengdu University of Traditional Chinese Medicine, Chengdu 611137, China; guoyurou@stu.cdutcm.edu.cn (Y.-R.G.); zhongwenxiao@stu.cdutcm.edu.cn (W.-X.Z.)

**Keywords:** nano–liposome, curcumin, tetrandrine, physicochemical properties, zebrafish, anti–tumor effects

## Abstract

(1) Background: Curcumin (CUR) and tetrandrine (TET) are natural compounds with various bioactivities, but have problems with low solubility, stability, and absorption rate, resulting in low bioavailability, and limited applications in food, medicine, and other fields. It is very important to improve the solubility while maintaining the high activity of drugs. Liposomes are micro–vesicles synthesized from cholesterol and lecithin. With high biocompatibility and biodegradability, liposomes can significantly improve drug solubility, efficacy, and bioavailability. (2) Methods: In this work, CUR and TET were encapsulated with nano–liposomes and g DSPE–MPEG 2000 (DP)was added as a stabilizer to achieve better physicochemical properties, biosafety, and anti–tumor effects. (3) Results: The nano–liposome (CT–DP–Lip) showed stable particle size (under 100 nm) under different conditions, high solubility, drug encapsulation efficiency (EE), loading capacity (LC), release rate in vitro, and stability. In addition, in vivo studies demonstrated CT–DP–Lip had no significant toxicity on zebrafish. Tumor cytotoxicity test showed that CT–DP–Lip had a strong inhibitory effect on a variety of cancer cells. (4) Conclusions: This work showed that nano–liposomes can significantly improve the physical and chemical properties of CUR and TET and make them safer and more efficient.

## 1. Introduction

Curcumin (CUR), a hydrophobic polyphenol compound, was first isolated from *Curcuma longa* L. in 1815 [1]. Studies have revealed its various biological and pharmacological effects [2,3], including anti–oxidation, anti–inflammatory, anti–cancer, and anti–arthritis. Owing to its bioactive properties, CUR is widely used as herbal medicine in many Asian countries to prevent and treat different types of diseases [4], such as skin diseases, chronic kidney disease, and rheumatoid arthritis. However, the biological applications of CUR were severely limited by its defects, such as low solubility, poor stability, low absorption rate, easy conversion into glucuronic acid, sulfonic acid, and other complexes in the intestine, fast metabolism, and short half–life. These shortages lead to its low bioavailability, which limits its application in the field of food and medicine [5]. Tetrandrine (TET) is a dibenzyl isoquinoline alkaloid extracted from *Stephania tetrandra* [6]. Modern pharmacological research showed that it has anti–inflammatory, anti–cancer, hypoglycemic, anti–fibrosis, and anti–hypertension [7,8]. It is used as an analgesic or to treat asthma, cancer, dysentery, fevers, and other diseases [9,10]. However, similar to CUR, the hydrophobicity of TET is also a major obstacle that limited its bioavailability and clinical application [6]. At the same time, some studies have shown that TET has side effects, such as hepatotoxicity and gastrointestinal tract after administration [8,9]. CUR has been reported in many studies for its alleviation of side effects caused by chemotherapy drugs [3]. Therefore, we chose to use these two natural drugs together.

Therefore, how to modulate the solubility and dissolution rate of bioactive but poorly water–soluble drugs and improving their bioavailability were crucial. In recent years, nanotechnology–based approaches are founded to increase the solubility of drugs. Nanoscale particles are more suitable for entering the blood circulation and giving full play to the curative effect of drugs [11]. Currently, nanotechnology researchers are putting effort into the development of new types of liposomes, polymer micelles, polymer nanoparticles, solid lipid nanoparticles, magnetic nanoparticles, albumin nanoparticles, and nano–sponges [12]. Among all, liposome delivery is considered to be very effective [13]. Liposomes are microvesicles synthesized from cholesterol and lecithin, have high biocompatibility and biodegradability, and have been widely used in cancer treatment [14]. Liposomes can encapsulate hydrophilic and lipophilic drugs simultaneously, and they can prolong the drug release time with high drug loading capacity at the same time, provide site–specific targeting, and control drug release [15]. The slow release of drugs can reduce the incidence of potential side effects and enhance the protection of drug physiological conditions [16]. However, this delivery method may have some drawbacks to incorporating lipophilic molecules. Some scholars suggested increasing the content of phospholipids to support high drug concentration [17], but only to find that this method was not feasible enough to support their hypothesis. Then, some scholars used the double loading method to prepare lipophilic drugs into water–soluble complexes with other carriers as the aqueous phase of hydrated liposomes [18,19]. However, this relatively complicated method is more suitable for drugs that can be encapsulated by water–soluble carriers. Studies have shown that, compared with inorganic delivery carriers, liposomes are more compatible and softer, suggesting that they are easier to be modified for drug delivery systems [13]. Thus, there are gradually more studies focusing on the modification of liposome structure to improve the feasibility of drug loading and the stability of the preparation [20].

1,2–distearoyl–sn–glycerol–3–ethanolamine phosphate–N–[methoxy (polyethylene glycol) –2000] (DSPE-MPEG 2000, DP) is a long-lasting stabilizer (the structure is shown in Figure S1), has the advantages of prolonging drug circulation time and enhancing the accumulation of the drug in tumor sites [21]. Traditional liposomes are particularly easy to be captured by the endothelial reticular system, resulting in their rapid clearance and affecting the curative effect [22]. After liposomes are modified with DP, the spatial exclusion barrier is formed by the strong hydration of liposomes from other macromolecules and cells so that liposomes can avoid being captured by the endothelial reticular system [23]. In the preparation of liposomes, DP is embedded in the polar group of liposomes in the form of covalent bonds [24], which can promote the formation of vesicles, and reduce the size of nanoliposomes, but also prevent the leakage of insoluble drugs in the preparation process. Now, it has been developed as a common stabilizer for liposomes [25,26]. Thus, in this work, DP was used as the stabilizer for double–loaded liposomes. In our studies, we selected the thin–film hydration method to prepare liposomes as it was easy to use. We further investigated the effects of different proportions of DP on the physical and chemical properties of liposomes and studied the in vitro release and stabilization, hepatoxic, biosafety, and anti–cancer activity of the prepared nano–liposomes.

## 2. Results

### 2.1. The Physical and Chemical Properties of Liposomes

**Figure 1:**
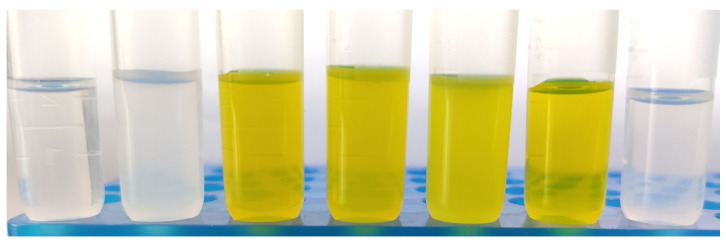
Optical photographs were taken for the appearance of liposomes. From left to right: Empty–DP–Lip, Empty–Lip, CT–DP–Lip, CT–TW80–Lip, CT–Lip, C–DP–Lip, and T–DP–Lip.

CT–DP–Lip, CT–Lip, CT–TW80–Lip, C–DP–Lip, T–DP–Lip, Empty–DP–Lip, and Empty–Lip were prepared successfully. Empty–Lip and Empty–DP–Lip were transparent solutions, the former was light gray, and blue fluorescence was reflected after the light irradiation; CT–Lip was yellow and opaque in the solution; CT–DP–Lip and CT–TW80–Lip were yellow, clear, and transparent (Figure 1).

The results of particle size, PDI, zeta potential, light transmittance, and turbidity of liposomes in each group were shown in Table S1. It can be seen from the table that the particle sizes of Empty–DP–Lip and Empty–Lip were 77.85 ± 3.64 nm and 187.88 ± 5.40 nm; the zeta potentials were −33.10 ± 3.43 mV and −21.21 ± 1.01 mV. The particle size of CT–DP–Lip and CT–Lip were 111.65 ± 1.34 nm and 255.74 ± 23.70 nm, and the zeta potentials were −30.45 ± 4.15 mV and 1.95 ± 0.62 mV. The comparison showed that the change range of particle size and potential of liposomes encapsulated with stabilizer DP was gentle, and the nanoparticles were still in a stable state of small size; however, after the drug was encapsulated by liposomes without stabilizer, the particle size and potential changed greatly, and the potential was converted from negative charge to positive charge. After replacing DP with Tween 80, the particle size of CT–TW80–Lip was 127.49 ± 4.07 nm, and the potential was −9.13 ± 6.90 mV.

The conductivity of CT–DP–Lip, CT–TW80–Lip and CT–Lip are 14.85 ± 0.86 μs/cm, 152.53 ± 2.63 μs/cm and 123.16 ± 6.37 μs/cm. It is suggested that the drugs in CT–DP–Lip were still encapsulated in liposomes in a molecular state, and there may be more ionic drugs in the latter two liposomes. The transmittance and turbidity of CT–DP–Lip, CT–TW80–Lip and CT–Lip are 83.94 ± 0.62% and 0.175 ± 0.007 cm^−1^, 76.94 ± 0.11% and 0.262 ± 0.001 cm^−1^, 0.47 ± 0.01% and 5.351 ± 0.016 cm^−1^. The greater the transmittance, the smaller the turbidity, indicating that the less insoluble suspended particles in the solution, the clearer the solution. On the contrary, it indicates that there are more insoluble suspended particles in the solution, and aggregation is easy to occur.

### 2.2. The EE and LC of CUR and TET

The CUR and TET contents in liposomes were measured as shown in Table S2. The EE and LC of CUR in CT–Lip were 48.91 ± 0.42% and 1.14 ± 0.01%; that of TET were 62.76 ± 1.01% and 1.47 ± 0.04%. When DP was added, the EE and LC of CUR were increased to 81.02 ± 2.70% and 1.89 ± 0.06%; that of TET were up to 89.51 ± 1.78% and 2.09 ± 0.04%. It is proved that DP as a stabilizer can significantly improve the LC and EE of the drug in liposomes.

### 2.3. Effect of DP Ratio on Drug–Loaded Liposomes

**Figure 2:**
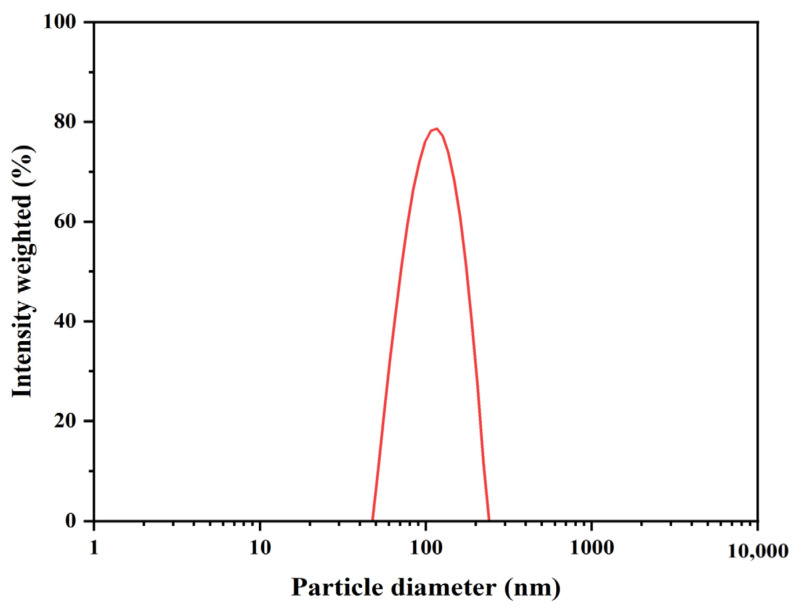
The particle size distribution of CT–DP–Lip.

DP has a long–chain shape. It could be embedded around the periphery of liposomes to prevent the leakage of drugs in liposomes during storage so that the particle size distribution of liposomes can not only maintain a small size but also maintain the stability of liposomes for a long time. The effect of the proportion of DP on drug–carrying liposomes was shown in Table S3. With the increase of the proportion of DP, the particle size and PDI decreased, and the potential increased, indicating that the CT–DP–Lip was more and more stable. Therefore, in the follow–up experiment, we set the dosage of DP in the preparation as 50%, and the particle size distribution is shown in Figure 2.

### 2.4. The FT–IR Spectrum

**Figure 3:**
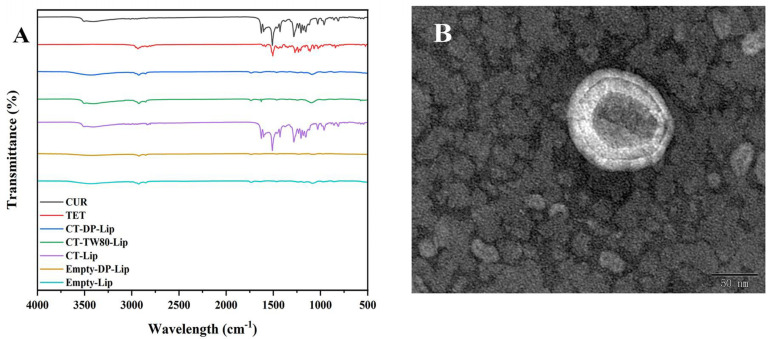
The Characterization diagram of liposomes. (**A**): The FT–IR spectrum of each group; (**B**): The TEM spectrum of CT–DP–Lip.

The FT–IR spectrum can verify whether liposomes successfully encapsulated drugs. The results were shown in Figure 3A. Empty–DP–Lip and Empty–Lip spectra were similar, indicating that the addition of DP had no significant change in the structure of liposomes. There are many characteristic absorption peaks in CUR: Stretching vibrations of phenolic hydroxyl, C=O, and aromatic C–O at 3503 cm^−1^, 1625 cm^−1^, and 1278 cm^−1^, respectively; Bending vibration of olefin C–H at 1429 cm^−1^. Similarly, in TET: The stretching vibration absorption peak of C–H at 2935 cm^−1^; 1607 cm^−1^, 1583 cm^−1^, and 1505 cm^−1^ are the stretching vibration absorption peaks of the benzene ring skeleton. The spectra of CT–DP–Lip and Empty–DP–Lip were similar, the characteristic absorption peaks of CUR and TET were masked, representing the formation of CT–DP–Lip, and the drug exists inside. In the spectra of CT–Lip, there were some characteristic absorption peaks of CUR. It is speculated that the lack of stabilizers may lead to the self–leakage of drugs. Although CT–TW80–Lip was similar to CT–DP–Lip, some studies had shown that [27], in addition to the interaction with liposomes, the drug is also solubilized in the micelles of TW80, which is consistent with the preliminary analysis results of the conductivity.

### 2.5. The TEM Spectrum

The appearance and morphology of CT–DP–Lip were observed by TEM, as shown in Figure 3B. It could be clearly seen in the figure that the nanoparticles showed a bimolecular layer with complete morphology, a smooth and flat surface, which was quasi–circular, which was consistent with the results of particle size analysis. At the same time, no obvious drug molecular particles were found, which echoed the FT–IR results, indicating that CUR and TET were successfully encapsulated in liposomes. The TEM images of other liposomes were shown in Figure S2.

### 2.6. The CR of Drugs in CT–DP–Lip and CT–Lip In Vitro

**Figure 4:**
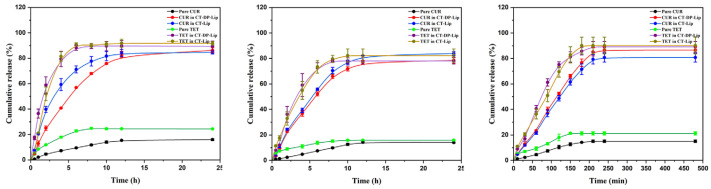
The cumulative release of CUR and TET in different media in vitro. The media were shown as: artificial gastric juice (pH 1.2, left), artificial intestinal fluid (pH 6.8, medium), and phosphate buffer solution (pH 7.4, right).

Figure 4 showed the CR of CUR and TET in different media. As shown in the figure, the CR curves of CUR and TET were significantly different between CT–DP–Lip and CT–Lip in different release media. At the same time, the release rate of the drug in the liposomes was significantly higher than pure CUR and TET, where CT–Lip was released higher than CT–DP–Lip. It is suggested that the preparation could significantly increase the CR of drugs in vitro. In addition, according to the characteristics of liposomes, they could release drugs slowly. It could be seen from Figure 4 that after adding DP, the drug release time was longer and longer, suggesting that liposomes added with stabilizer could not only increase the CR of drugs but also make drugs release slowly. This process increased the half–life of drugs and reduced the burden on metabolic organs.

### 2.7. Stability Investigation of CT–DP–Lip

**Figure 5:**
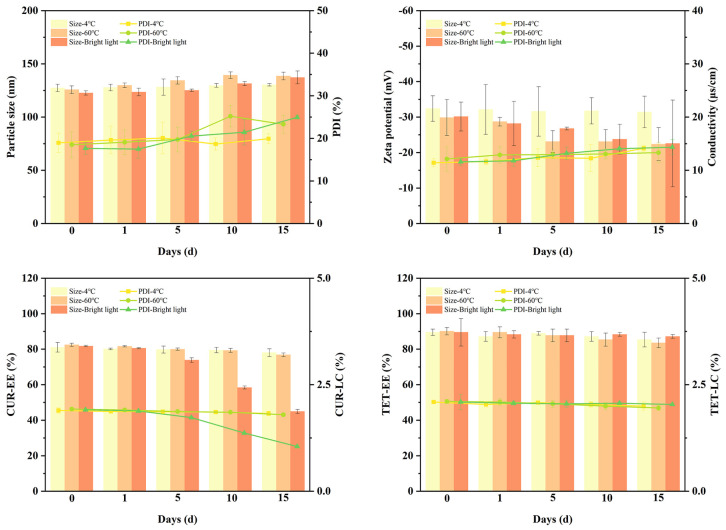
The particle size, PDI, EE, and LC of CT–DP–Lip in different environments.

Figure 5 showed the stability results of CT–DP–Lip in different environments. With the increase of storage time, the appearance of 4 °C and 60 °C groups changed little, which were the clear yellow solutions. In the bright light irradiation group, the color of the solution changed to yellowish. The various parameters of CT–DP–Lip remained almost the same in the 4 °C groups, while in the 60 °C and bright light irradiation groups they changed a lot. The EE and LC of CUR and TET changed obviously in the 60 °C groups, speculating that the high temperature accelerated the movement of drug molecules and leaked from liposomes, resulting in the decrease of EE and LC. It is worth noting that the EE and LC of TET did not change significantly in the bright light irradiation group, but that of CUR changed dramatically. The EE of CUR decreased from 81.73 ± 0.38% to 44.93 ± 1.16%, and the LC of CUR decreased from 1.91 ± 0.01% to 1.05 ± 0.03%, indicating that the content of CUR decreased significantly after stimulated by bright light irradiation, which was consistent with the results of other researchers [28] and the change of the appearance color of the CT–DP–Lip. It is pointed out that CUR will decompose with the extension of irradiation time, so the CT–DP–Lip should be stored in a dark and cool environment.

### 2.8. Zebrafish Liver Toxicity Test

#### 2.8.1. The Morphological Development of Zebrafish

**Figure 6:**
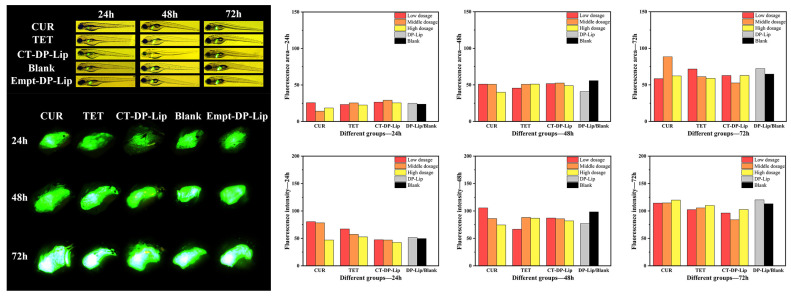
The morphological images of zebrafish and fluorescence images of zebrafish liver in the high dose group (**left**). The fluorescence area and intensity of each group in different time periods (**right**).

Administrating accordingly from the experiments above, zebrafish in each group were collected, and the liver of zebrafish larvae was photographed by a fluorescence microscopic imaging system and an inversion microscopic imaging system. The results were shown in Figure 6. Zebrafish morphological analysis showed that zebrafish in each group developed well without obvious deformity (*p* > 0.05), indicating that pure drugs, CT–DP–Lip, and Empty–DP–Lip had no significant effect on zebrafish morphological development after 72 h incubation.

#### 2.8.2. The Fluorescence Area and Intensity of Zebrafish Liver

The light source was adjusted to fluorescence mode, and the results were shown in Figure 6. It can be seen from the figure that the fluorescence images of zebrafish liver in different administration groups were clearly visible, and the fluorescence intensity and area of the liver increased with the increase of culture time, indicating that the zebrafish grew healthily (*p* > 0.05).

The fluorescence intensity and area of zebrafish liver in each group were analyzed by Image J software, and the results were shown in Figure 6. With the extension of experiment time, the fluorescence intensity and area of zebrafish liver in different treatment groups increased. It was found that there were significant differences in liver fluorescence intensity and area in each group at different time periods (*p* < 0.05). There was no significant difference in liver fluorescence intensity between pure CUR and the blank group (*p* > 0.05). In the pure TET group, the liver fluorescence intensity in the low–dose group was significantly lower than that in the medium and high–dose groups and the blank group (*p* < 0.05). The above showed that CUR and TET will have a certain impact on the liver area and intensity of zebrafish. Similarly, we also investigated the effects of C–DP–Lip and T–DP–Lip on the fluorescence area and intensity of zebrafish liver (Figure S3). The results showed that the high concentration of C–DP–Lip could enhance the fluorescence intensity of zebrafish liver, while the high concentration of T–DP–Lip may affect the development of zebrafish liver. However, CT–DP–Lip had no significant effect on the fluorescence intensity and area of zebrafish liver, which showed that the CT–DP–Lip had no significant effect on zebrafish liver and could eliminate the side effects of CUR and TET as well. This indicates the biosafety of CT–DP–Lip. In addition, during the experiment, fluorescence signals appeared in the eyes and blood vessels of zebrafish in the high–dose group and CUR and CT–DP–Lip group, which was consistent with the research of Liu et al. [29]. It suggested that CUR has fluorescence characteristics and can be used as a fluorescent indicator and tracker for further studies.

### 2.9. Anti–Tumor Effect In Vitro

**Figure 7:**
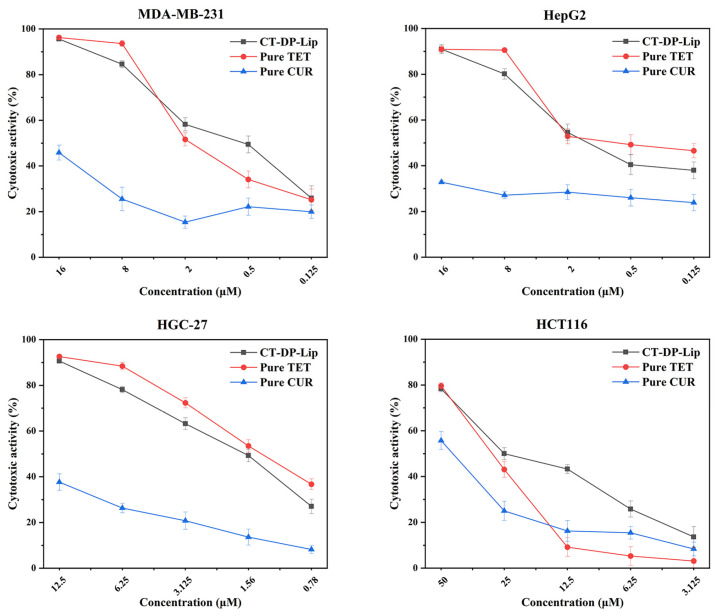
The cytotoxicity of CT–DP–Lip in MDA–MB–231 cells, HepG2 cells, HGC–27 cells, and HCT116 cell lines in vitro.

In this work, we used the MTT assay to detect the cytotoxicity of CT–DP–Lip in MDA–MB–231, HepG2, HGC–27, and HCT116 cell lines in vitro. The preliminary experiment found that CUR did not show obvious cytotoxicity to four kinds of cells below 12.5 μM, and the cell inhibition rate was less than 50%. Therefore, the concentration of CT–DP–Lip in the follow–up cytotoxicity test was calculated by TET content. The MTT results were shown in Figure 7. The IC_50_ values of CT–DP–Lip in MDA–MB–231, HepG2, HGC–27, and HCT116 cells were 0.692, 0.647, 1.816, and 18.084 μM (in terms of TET content), respectively. CT–DP–Lip was significantly cytotoxic to four cell lines and showed a wide range of antitumor effects. Among them, the cytotoxicity to MDA–MB–231 and HepG2 cells was the strongest, followed by HGC–27 cells, and the toxicity to HCT116 cells was relatively low, indicating that the CT–DP–Lip had certain selectivity and sensitivity among the carcinoma cells. The in vitro results of CT–DP–Lip, C–DP–Lip, T–DP–Lip, CUR, and TET in MDA–MB–231 cell lines were attached in Table S4. It showed that the IC_50_ values of C–DP–Lip and T–DP–Lip were much higher than those of CT–DP–Lip. It is speculated that there are more binding sites in the bilayer of the drug due to the separate encapsulation of the drug, and it takes longer for the drug to be fully released and uptake into the cells.

## 3. Discussion

**Figure 8:**
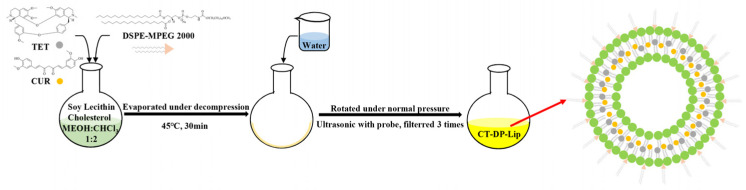
The schematic diagram of the preparation of CT–DP–Lip.

CUR and TET are natural products with good biological activity but poor solubility, resulting in their low bioavailability, which limits their application in various fields, such as food and medicine [30,31]. Nanoliposomes with good biocompatibility can significantly increase the solubility and bioavailability of poorly water–soluble drugs like CUR and TET [32]. However, liposomes still have poor stability and self–leakage for drugs with special physical and chemical properties. Therefore, modifiers are selected to protect the stability of liposomes [33]. DP is a widely used stabilizer in liposomes [34]. The experimental results showed that the addition of DP could not only significantly improve the particle size distribution of CT–DP–Lip but also significantly improve the EE and LC of CUR and TET in liposomes. It is speculated that the possible mechanism is that the “head” in the DP structure is embedded in the peripheral structure of the liposome to prevent drug leakage during preparation and storage and ensure the stability of drugs in the liposome (Figure 8).

The characterization was conducted to determine the successful synthesis of the liposomes followed by an analysis of drug binding forms and structural characteristics in the formation process. This provided us with the data basis for the subsequent combination of insoluble drugs and nano–liposomes. The results of FTIR (Figure 3A) were consistent with previous studies [35,36], DP will not affect the structure of liposomes after adding liposomes. TEM results (Figure 3B) also confirmed this point. The liposome was quasi–circular as a whole, the surface was smooth and complete, the structure was not damaged, and the phospholipid bilayer was clearly visible. The electrical conductivity showed that most of the drugs in CT–DP–Lip were encapsulated in liposomes in a molecular state. The turbidity showed that there were fewer suspended particles in CT–DP–Lip, the light transmittance was good, and the solution was uniform and stable.

Zebrafish have a strong reproductive ability and a short experimental cycle, it is one of the classical models for rapid screening of drug safety [37]. The high and low–dose CUR groups may promote the liver development of zebrafish; low–dose TET will have a certain impact on the liver area and intensity of zebrafish (Figure 6). The CT–DP–Lip had no significant effect on the fluorescence intensity and area of zebrafish liver, which showed that CT–DP–Lip had no obvious effect on zebrafish liver and eliminated the hepatotoxic effects of CUR and TET as well. This indicated that the CT–DP–Lip was relatively safe. In addition, during the experiment, fluorescence spots appeared in the eyes and blood vessels of zebrafish in the high–dose group and CUR and CT–DP–Lip group, which is consistent with the research of Liu et al. [29], suggesting that CUR has fluorescence characteristics and can be used as a fluorescent indicator and tracker for the next experiment.

The cytotoxicity of CUR and TET were lower than the CT–DP–Lip. CUR, especially, showed no significant cytotoxicity in four carcinoma cell lines. Although TET showed relatively strong cytotoxicity, its poor solubility, however, was still a huge obstacle. The cytotoxicity of CT–DP–Lip to MDA–MB–231 cells at a low dose was significantly higher than either pure TET or CUR, indicating that the combination of the two could significantly enhance the antitumor efficacy (*p* < 0.05) while also maintaining relative biosafety.

## 4. Materials and Methods

### 4.1. Materials

CUR and TET (98% purity grade) were purchased from Xi’an XiaoCao Botanical Development Co., Ltd (Xi’an, China). Soybean lecithin, cholesterol, and DSPE–MPEG 2000 were purchased from A. V. T. (Shanghai, China) Pharmaceutical Co., Ltd. Trypsin (1:250) was purchased from Solarbio (Beijing, China). Pepsin (1:10,000) was purchased from Sigma (Shanghai, China). High–performance liquid chromatography (HPLC)–grade acetic acid and triethylamine (≥99.8% purity grade) were purchased from Chengdu Kelong Chemical Co., Ltd (Chengdu, China). HPLC–grade methanol and acetonitrile (≥99.8% purity grade) were purchased from Thermo Fisher Scientific (Shanghai, China). Dulbecco’s modified Eagle’s medium (DMEM, pH 7.5) and HQ fetal bovine serum (FBS) were purchased from TransGen Biotech Co., Ltd (Beijing, China). Deionized water was produced by ULUPURE laboratory ultrapure water machine (Chengdu, China). All other chemicals were of analytical grade and utilized without further purification.

### 4.2. Animals and Cells

SPF–grade liver fluorescent transgenic zebrafish Tg (lfabp10: EGFP) was purchased from the National Zebrafish Resource Center. MDA–MB–231, HepG2, HGC–27, and HCT116 cell lines were provided by the State Key Laboratory of Southwestern Chinese Medicine Resources.

### 4.3. Preparation of Liposomes

The preparation method adopted the thin–film hydration method [38]. The experimental protocol was adjusted as followed: Soybean lecithin, cholesterol, DP, CUR, and TET were placed in a round bottom flask. Methanol and chloroform were added as solvents. Next, the organic solvent was dried slowly, and a yellow, transparent, and uniform film was formed. UP water was added, then ultra–sonicated for 5 min, and finally extruded and filtered three times to remove the leftover CUR and TET. The CUR–TET liposomes now homogenized the particle size, and ready to be modified as CT–DP–Lip. Similarly, CT–Lip was obtained without DP; Empty–DP–Lip was prepared without CUR and TET; And Empty–Lip was obtained without adding DP, CUR, and TET. The EE (%) and LC (%) can be calculated according to the following Formulas (1) and (2) [39].

(1) $$\mathrm{EE}=\frac{\mathrm{The}\;\mathrm{weight}\;\mathrm{of}\;\mathrm{drug}\;\mathrm{measured}}{\mathrm{The}\;\mathrm{weight}\;\mathrm{of}\;\mathrm{drug}\;\mathrm{added}}\times 100$$

(2) $$\mathrm{LC}=\frac{\mathrm{The}\;\mathrm{weight}\;\mathrm{of}\;\mathrm{drug}\;\mathrm{measured}}{\mathrm{The}\;\mathrm{weight}\;\mathrm{of}\;\mathrm{all}\;\mathrm{material}}\times 100$$

### 4.4. Determination of Physical and Chemical Properties

One milliliter sample was taken to measure particle size, polydispersity index (PDI), zeta potential, and conductivity respectively by Particle Analyzer. According to the method of Deng et al. [40], the light was emphasized to 20 mW, and the transmission wavelength was adjusted to 680 nm. The light transmittance of the sample was measured, and the turbidity (cm^−1^) was calculated according to the following Formula (3). All samples were diluted to the same multiple and measured at room temperature, and the determination was repeated three times.

(3) $$\mathrm{Turbidity}=\frac{D}{L}\cdot \mathrm{In}\left(\frac{I_{0}}{I}\right)=\frac{D}{L}\mathrm{In}(1/T)$$

D: Dilution factor; L: Optical path length; I0: Intensity of light source passing through calibrator; I: Intensity of light source passing through the sample; T: Transmittance.

### 4.5. Effect of DP Ratio on Drug–Loaded Liposomes

To validate the stabilizer DP, the non–ionic surfactant Tween 80 and different proportions of DP (5, 10, 15, 20, 30, and 50%) were tested for their relevance to particle size, PDI, and zeta potential of liposomes. These comparison studies were conducted as pre–trial experiments. CT–TW80–Lip was obtained by replacing DP with the same amount of Tween–80, while other experimental parameters remained the same.

### 4.6. Determination of Release In Vitro

According to the study by Chen et al. [25], the dialysis bag method could be used for mimicking liposome release in vitro. The pre–trial experiment indicated the solubility of the CUR and TET was significantly improved in CT–DP–Lip (Figure S4). Therefore, pure drugs need to add cosolvent (Tween 80) to completely dissolve in the release medium. Ten milliliters of artificial gastric juice (containing pepsin) (pH 1.2), artificial intestinal juice (containing pancreatin) (pH 6.8), and phosphate buffer (with 0.3% Tween 80, *v*/*v*) (pH 7.4) were the dissolution mediums, respectively. One–milliliter samples were respectively put into the dialysis bag, the opening was closed with a sealing clip and checked for leakage. The sealed dialysis bags were placed in 10 mL of three different release media, the temperature was adjusted to 37 ± 0.5 °C, and rotation was set at 100 rpm. After each group is sampled, it is necessary to supplement the corresponding medium with the same temperature and volume. The cumulative release (CR, %) in this time period is calculated according to the following formula (4).

(4) $${\mathrm{CR}}_{n}=\frac{m_{1}{+m}_{2}{+m}_{3}+\ldots +m_{n}}{\mathrm{The}\;\mathrm{weight}\;\mathrm{of}\;\mathrm{drug}\;\mathrm{added}}\times 100$$

Note: m_1_ is the drug amount measured in the first time period, and so on.

### 4.7. Stability Test

This study investigated the stability of CT–Lip and CT–DP–Lip by dramatically changing the storage environment. This work was designed to explore the rationality of the preparation process with stability and provide information for storage conditions during the preparation [41]. Three environmental factors were set up: 4 °C, 60 °C, and bright light irradiation (4 500 ± 500 Lx). Part of CT–DP–Lip was put into the glass test tube and placed in the corresponding conditions for 15 days. Every five days, 2 mL of solution was taken out (day 0, 1, 5, 10, and 15) for physical changes observation. The particle size, PDI, zeta potential, conductivity, EE, and LC were measured according to the early–stage method.

### 4.8. Zebrafish Liver Toxicity Test

Three–month–old liver fluorescent transgenic zebrafish Tg (lfabp10: EGFP) was randomly selected. The actual experimental conditions were carried out according to the research of Liu and Song et al. [42,43]. All experiments were conducted in the accordance with legal regulation and ethical approval from the Institutional Animal Ethics Committee of the Chengdu University of TCM.

The zebrafish were divided into high, medium, and low dose groups (CT–DP–Lip, pure CUR, and TET), Empty–DP–Lip, and Black (0.1% DMSO solution) test groups according to the administered dose. The morphology of zebrafish and their livers were observed and photographed by fluorescence microscopy and inversion microscopy imaging system [44]. Finally, the fluorescence area and intensity of zebrafish liver in the photos were analyzed by Image J software.

### 4.9. Cell Culture

The target cells were incubated in DMEM supplemented with 10% TransSerum^TM^ HQ FBS and 1% penicillin–streptomycin at 37 °C in a humidified atmosphere containing 5% CO_2_.

### 4.10. Anticancer Activity Assay In Vitro

The target cells in the logarithmic growth period were taken and inoculated in 96 well plates according to 4000 per well, with a 100 μL culture medium per well (10% FBS, 1% penicillin–streptomycin). After 24 h of continuous culture, removing the original medium, a 100 μL predetermined concentration of medium (1% FBS and 1% penicillin–streptomycin) containing CT–DP–Lip, CUR, and TET were added to the designated well. After 48 h of culture, 20 μL 3–(4,5–dimethylthiazol–2–yl)–2,5–diphenyltetrazolium bromide (MTT) stock solution was administered to each well and incubated for 4 h. Then, the medium containing MTT was completely removed and 150 μL DMSO was added to each well. Use a microplate reader to read the absorbance value at 490 nm. The blank control group was not given medicine. The other operations were the same as above. Each column was administered for different concentrations (each concentration was repeated 6 times). Cytotoxicity (%) was calculated according to the following Formula (5).

(5) $$\mathrm{Cytotoxicity}=\frac{\mathrm{The}\;\mathrm{OD}\;\mathrm{value}\;\mathrm{of}\;\mathrm{blank}\;\mathrm{group}\text{–}\mathrm{The}\;\mathrm{OD}\;\mathrm{value}\;\mathrm{of}\;\mathrm{administration}\;\mathrm{group}}{\mathrm{The}\;\mathrm{OD}\;\mathrm{value}\;\mathrm{of}\;\mathrm{blank}\;\mathrm{group}}\times 100$$

### 4.11. Characterization Determination

To verify the successful preparation of liposomes, we used FTIR to measure the mid–infrared spectrum of liposomes. The sample was prepared by the potassium bromide tablet pressing method, and the scanning wavelength was 4000–400 cm^−1^. The appearance and morphology were presented in the form of transmission electron microscope pictures (TEM) after negative staining. The sample was placed on a common copper net and negatively stained with 1% (*w*/*v*) phosphotungstic acid, and the appearance was observed and photos were taken.

## 5. Conclusions

In the present work, we proposed a novel approach to enhance the low water solubility and poor bioavailability of CUR and TET by adding DP followed by a liposomal drug delivery system. The size, morphology, physical and chemical properties, EE, LC, solubility, stability, release in vitro, safety, and anti–tumor effect of MDA–MB–231, HepG2, HGC–27, and HCT116 cell lines in vitro were evaluated consequently. As a new and safe drug delivery system, liposomes can improve the solubility of CUR and TET, break the restrictions on their clinical application due to their chemical properties and increase anticancer activity. This work can inspire the research of therapeutic nanoparticles with liposomal drug delivery systems which enhance the bioavailability of poorly water–soluble drugs and further validate the feasibility of CUR as self–monitoring molecules for tumor–targeting therapy.

## Data Availability

The data presented in this study are available upon request from the corresponding author.

## Supplementary Materials

The following supporting information can be downloaded at: https://www.mdpi.com/article/10.3390/ijms23126858/s1.
