# Supplementary material for: Nano–Liposomes Double Loaded with Curcumin and Tetrandrine: Preparation, Characterization, Hepatotoxicity and Anti–Tumor Effects
Source: Int J Mol Sci. 2022 Jun 20;23(12):6858. doi: 10.3390/ijms23126858 (PMC9224699; doi:10.3390/ijms23126858)
Supplement: Supplementary file 1 [file ijms-23-06858-s001.zip › ijms-1755654-supplementary.pdf]

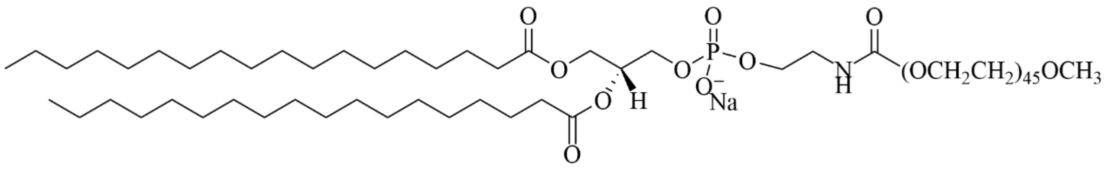

Figure S1. The structure of DSPE–MPEG 2000.

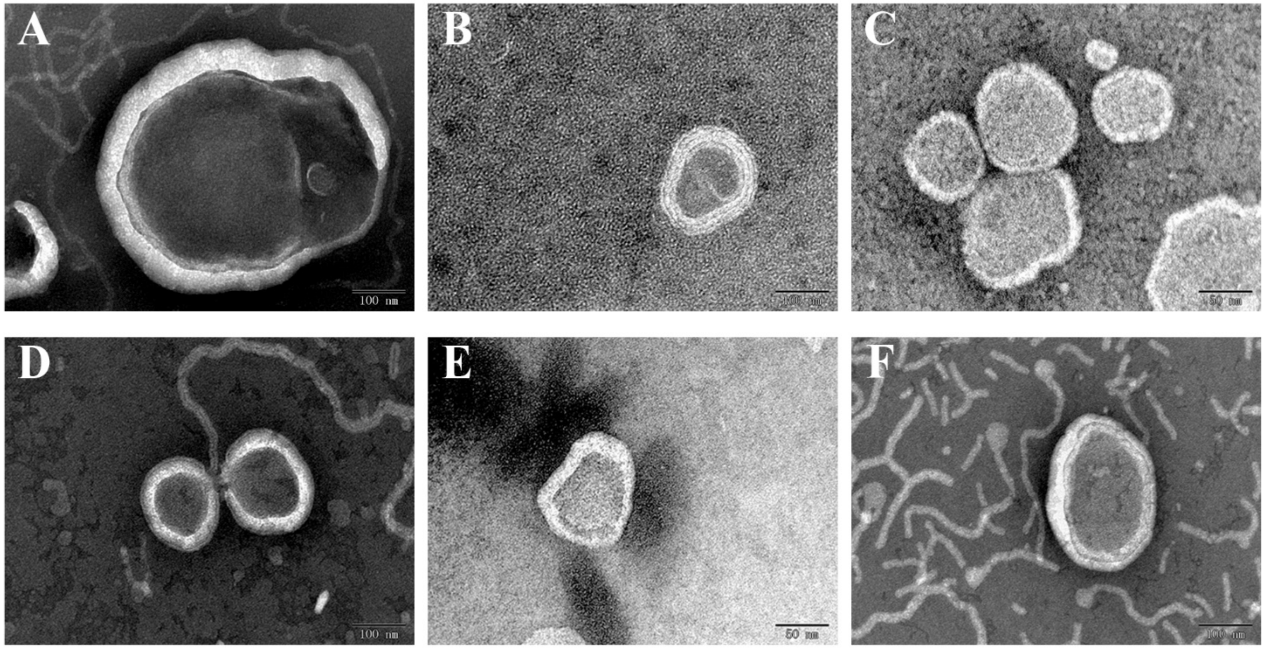

Figure S2. The TEM spectrum of A) Empty–Lip, B) DP–Lip, C) CT–TW80–Lip, D) C–DP–Lip, E) T–DP–Lip, F) CT–Lip.

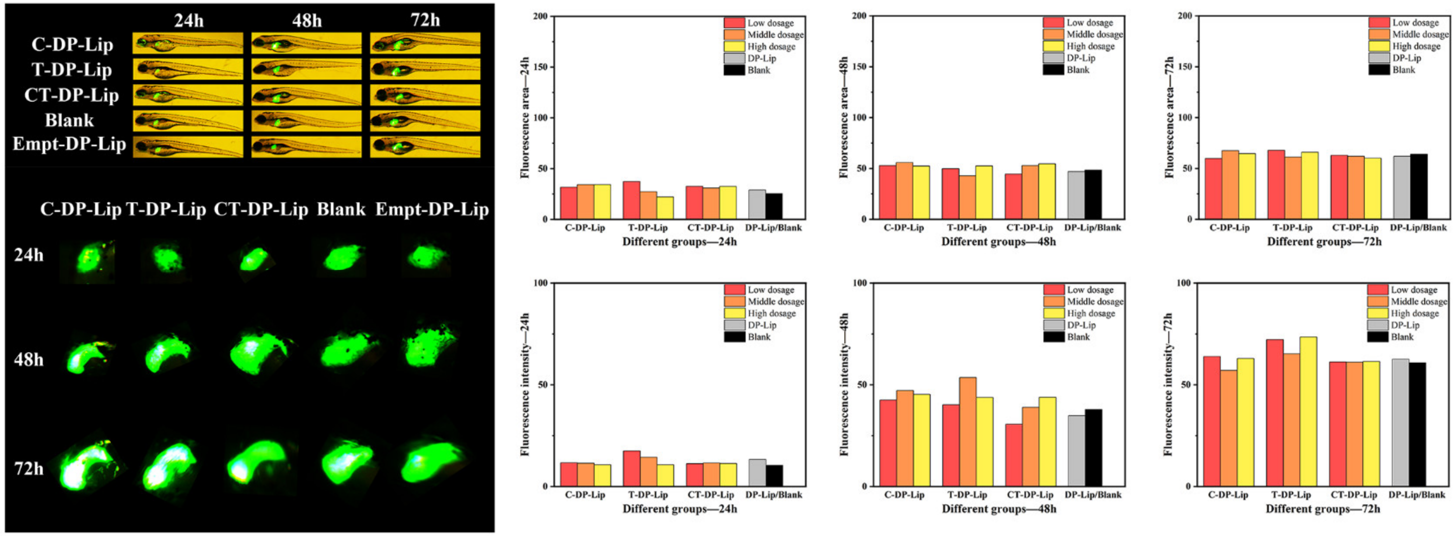

Figure S3. The fluorescence area and intensity of zebrafish liver of CT–DP–Lip, C–DP–Lip, T–DP–Lip and DP–Lip groups in different time periods.

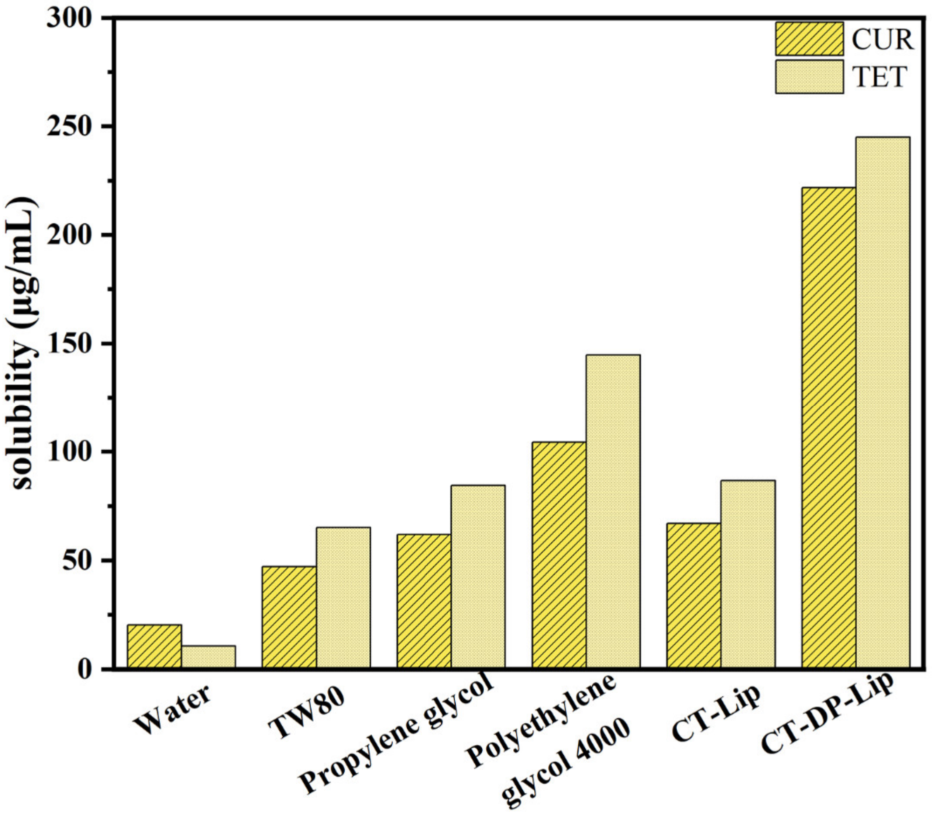

Figure S4. The solubility of CUR and TET in different solvents.

Table S1. The results of particle size, PDI, zeta potential, transmittance and turbidity of liposomes in each group

Name	Mean particle size (nm)	PDI (%)	Zeta potential (mV)	Conductivity (μs/cm)	Transmittance (%)	Turbidity (cm^{-1})
CT–DP–Lip	111.65 ± 1.34	21.59 ± 2.60	–30.45 ± 4.15	14.85 ± 0.86	83.94 ± 0.62	0.175 ± 0.007
CT–TW80–Lip	127.49 ± 4.07	15.32 ± 2.48	–9.13 ± 6.90	152.53 ± 2.63	76.94 ± 0.11	0.262 ± 0.001
CT–Lip	255.74 ± 23.70	26.74 ± 1.47	1.95 ± 0.62	123.16 ± 6.37	0.47 ± 0.01	5.351 ± 0.016
C–DP–Lip	138.08 ± 2.77	21.83 ± 2.10	–47.97 ± 0.95	17.33 ± 0.58	78.03 ± 0.12	0.25 ± 0.001
T–DP–Lip	98.58 ± 1.24	23.30 ± 0.60	–21.73 ± 0.23	44.33 ± 0.58	71.67 ± 0.06	0.33 ± 0.001
Empty–DP–Lip	77.85 ± 3.64	14.81 ± 1.53	–33.10 ± 3.43	10.16 ± 0.40	86.08 ± 0.65	0.150 ± 0.008
Empty–Lip	187.88 ± 5.40	23.98 ± 1.88	–21.21 ± 1.01	122.73 ± 3.06	11.13 ± 0.35	2.196 ± 0.031

Note: Data are the mean ± standard deviation of three replicates.

Table S2. The encapsulation efficiency and loading content of CUR and TET in CT–Lip and CT–DP–Lip

Name	EE (%)		LC (%)	
	CUR	TET	CUR	TET
CT–DP–Lip	81.02 ± 2.70	89.51 ± 1.78	1.89 ± 0.06	2.09 ± 0.04
CT–Lip	48.91 ± 0.42	62.76 ± 1.01	1.14 ± 0.01	1.47 ± 0.04

Note: Data are the mean ± standard deviation of three replicates.

Table S3. The effect of the proportion of DP on drug–carrying liposomes

DP Proportion (%)	Mean particle size (nm)	PDI (%)	Zeta potential (mV)
5	289.07 ± 23.45	26.40 ± 0.35	–9.10 ± 6.89
10	272.27 ± 5.25	26.57 ± 4.76	–20.63 ± 0.72
15	255.37 ± 16.75	23.27 ± 1.36	–21.20 ± 1.04
20	127.49 ± 4.07	15.33 ± 2.48	–20.20 ± 0.70
30	111.64 ± 1.34	21.59 ± 2.60	–16.35 ± 2.92
50	97.41 ± 2.21	14.87 ± 0.23	–33.10 ± 3.46

Note: Data are the mean ± standard deviation of three replicates.

Table S4. The IC_{50} values of CT–DP–Lip, C–DP–Lip, T–DP–Lip, CUR and TET in MDA–MB–231 cell lines *in vitro* (μM)

Groups	DP–Lip	CUR	TET	C–DP–Lip	T–DP–Lip	CT–DP–Lip
IC_{50}	NS	19.103	1.120	12.426	3.764	0.692

Note: NS indicates that there is no obvious cytotoxicity and IC_{50} cannot be calculated.
